# Artefacts in Hysterectomies with a Special Focus on Vascular Pseudoinvasion

**DOI:** 10.3390/diagnostics14161833

**Published:** 2024-08-22

**Authors:** Sami Dagher, Mousa Mobarki, Celine Chauleur, Alexandra Papoudou-Bai, Michel Péoc’h, Georgia Karpathiou

**Affiliations:** 1 Pathology Department, University Hospital of Saint-Etienne, 42270 Saint-Etienne, France; sdagher@hotmail.fr (S.D.); michel.peoch@chu-st-etienne.fr (M.P.); georgia.karpathiou@chu-st-etienne.fr (G.K.); 2 Department of Basic Medical Sciences (Pathology), Faculty of Medicine, Jazan University, Jazan 45142, Saudi Arabia; 3 Gynecology and Obstetrics Department, University Hospital of Saint-Etienne, 42270 Saint-Etienne, France; celine.chauleur@chu-st-etienne.fr; 4 Pathology Department, Faculty of Medicine, University of Ioannina, 451 10 Ioannina, Greece; apapoudoubai@gmail.com

**Keywords:** manipulator, pseudoinvasion, emboli, vascular, laparoscopic

## Abstract

Background: Since the advent of laparoscopic hysterectomy, several studies have described artefacts, such as vascular pseudoinvasion, constituting potential pitfalls in the histological evaluation of these specimens. The use of an intrauterine manipulator is often suggested as the factor creating these artefacts. Objectives: To describe possible artefacts, such as vascular pseudoinvasion, myometrial clefts, and tumor cells in the lumen of the cervix, on the serosa, and in the tubal lumen, and to correlate them with clinical and pathological characteristics. Material and Methods: This is a retrospective monocentric study of 60 patients having been treated for benign (*n* = 27, 45%) or malignant (*n* = 33, 55%) uterine pathologies. Results: Vascular pseudoinvasion was found in 13 (22%) adenocarcinomas and in one (2%) benign uterine pathology. Clefts within the myometrium were observed in 16 (27%) uteri. Cells in the tubal lumen were observed in six (10%) hysterectomies. True vascular emboli were not correlated with the use of an intrauterine manipulator (*p* = 0.47) or the type of surgery (*p* = 0.21). Vascular pseudoinvasion was correlated with the presence of tumor cells in the lumen of the cervix (*p* = 0.013) and the presence of clefts in the myometrium (*p* < 0.001), but not with the other factors studied. Conclusions: Overall, in our series, we did not observe any statistical association between the use of an intrauterine manipulator and the presence of true emboli or vascular pseudoinvasion during hysterectomy in women with malignant or benign uterine pathologies. Vascular pseudoinvasion was also associated with the presence of other artefacts.

## 1. Introduction

For several years, total laparoscopic hysterectomy and robot-assisted hysterectomy have been developing, showing fewer postoperative morbidities, in particular less pain, faster recovery, shorter hospitalization, and an equivalent capacity to stage endometrial adenocarcinoma compared to total hysterectomy by abdominal laparotomy [1,2]. Nevertheless, some studies show that this laparoscopic technique could be the source of a greater number of positive peritoneal cytologies [3,4], and more emboli and artefacts [5] mimicking the dissemination of tumor cells. During this procedure, a pneumoperitoneum is made; an intrauterine manipulator is inserted into the endometrial cavity transvaginally, creating an increased intrauterine pressure which, according to some authors, could be responsible for the phenomenon of vascular pseudoinvasion [6] but also the displacement of tumor cells in the cervical lumen, in the tubal lumen, or even in slits inside the myometrium [7].

The first work describing uterine vascular pseudoinvasion was performed in 37 laparoscopic hysterectomies, carried out for benign and malignant uterine pathologies, showing tumoral epithelial cells in the vessels in 71% of tumoral specimens and the presence of endometrium in the vessels of 13% of non-tumor samples [6]. A second study compared laparoscopic hysterectomy to abdominal laparotomic hysterectomy performed for early-stage endometrial adenocarcinoma. The authors observed vascular emboli in 33% of laparoscopic hysterectomies, whereas no emboli were found in hysterectomies by laparotomy [8]. However, some studies did not find any statistically significant differences in the presence of emboli and pseudoemboli [9,10,11] between the two surgical techniques. Another study showed only a tendency for greater vascular pseudoinvasion in patients treated by the laparoscopic technique [12], while Krizova et al. showed that laparoscopic hysterectomy using an intrauterine manipulator was more often associated with vascular pseudoinvasion, epithelial cells in the tubal lumen, and cell-crushing artefacts, independently of benignity or malignancy [7]. The authors suggested that it is not the laparoscopy procedure that causes pseudoemboli, but the use of an intrauterine manipulator [7]. Another study did not show any difference in the proportion of vascular invasion between these two surgical techniques, but without distinguishing emboli or pseudoemboli [5]. It is worth mentioning that vascular pseudoinvasion has also been described in other tissues. Indeed, McLachlin et al. [13] reported a pseudoinvasion of the dysplastic squamous epithelium of the cervix into the vessels following the injection of lidocaine used before the procedure. Karpathiou et al. [14] observed this phenomenon in a patient treated by hysterectomy after endometrial ablation.

In this study, we wanted to see if there were more emboli, pseudoemboli, and other microscopic artefacts on laparoscopic with intrauterine manipulator hysterectomy than on laparotomic hysterectomy specimens, and to correlate them with clinicopathological parameters.

## 2. Materials and Methods

This is a retrospective study. The local ethics committee approved the study (Comité d’Ethique du CHU de Saint-Etienne, IRBN552021/CHUSTE, 17 March 2021). Hysterectomies for benign lesions (*n* = 27) were consecutive specimens, while for malignant lesions (*n* = 33), all endometrioid adenocarcinomas corresponded to a series previously studied, with microsatellite instability (MSI) status for some of them, in case this characteristic could influence vascular invasion [15]. Clinical and demographic data were extracted from electronic medical files. The type of surgery, tumor size, lymph node status, presence or absence of local recurrence, distant metastasis, and adjuvant treatment (brachytherapy, hormone therapy, or radiotherapy) were noted. Our standardized macroscopic examination protocol consists of inking of the different surgical limits, separation of the cervix from the uterine body, and opening of the uterine body along its anterior surface. The surgical specimen is then placed in 10% neutral buffered formalin for at least 24 h. All tumor slides were examined using Hematoxylin, Eosin and Saffron (HES) staining. The slides were examined simultaneously by three pathologists until final agreement. The diagnosis of endometrial adenocarcinoma was made according to the corresponding WHO classification [16]. The TNM was reassessed according to the 8th edition of the AJCC [17].

The histopathological criteria collected were: the histological type, the tumor grade, the presence or absence of tumor necrosis, the status of MMR proteins (followed by MSI testing), the presence or absence of true vascular emboli, the presence or absence of vascular pseudoinvasion, and the presence or absence of tumor cells in the lumen of the cervix, on the serosa, in the tubal lumen, and in the artefactual clefts in the myometrium. According to the 2020 WHO classification of gynecological tumors, grade 1 and grade 2 tumors were considered low-grade tumors, and grade 3 tumors were considered high-grade tumors.

**Figure 1:**
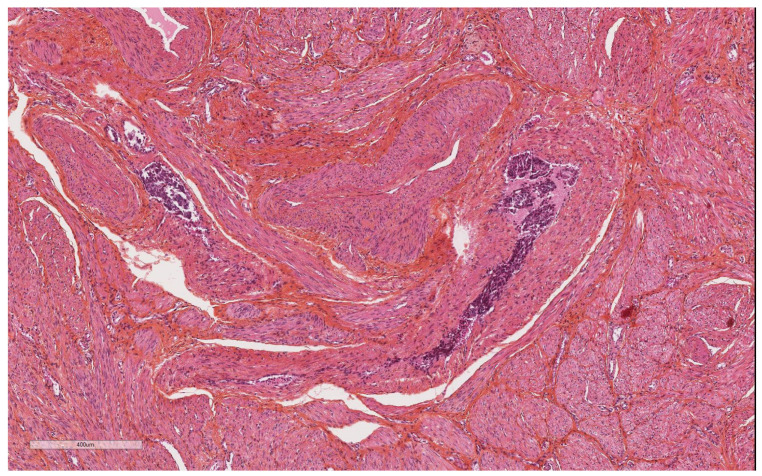
True vascular tumor emboli.

**Figure 2:**
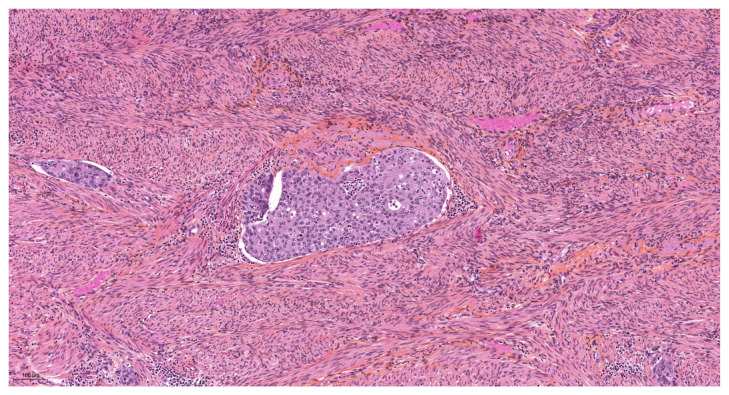
Vascular pseudoinvasion by tumor cells.

**Figure 3:**
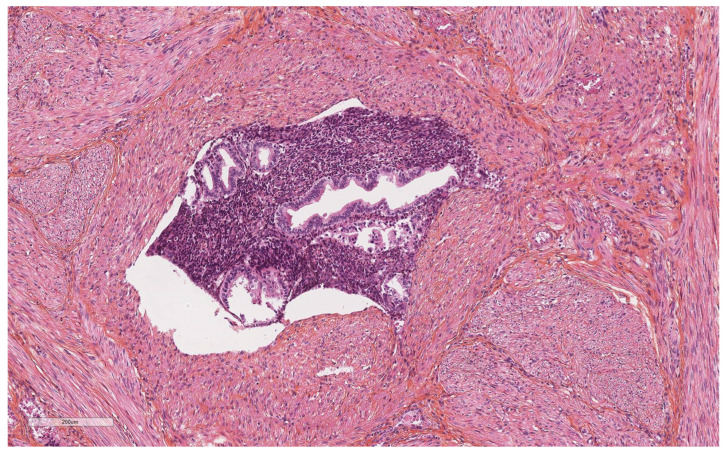
Vascular pseudoinvasion by normal endometrium.

More specifically for vascular evaluation, the size of the vessels involved (large vessels of the external third of the myometrium, or small vessels) and their nature (lymphatic, venous, or arterial) were reported. Their location in the anterior, posterior, or lateral uterine wall was noted. We used the morphological criteria of Folkins et al. [12] to define true vascular invasion. A true embolus was diagnosed (Figure 1) when there were cohesive, smooth-edged clusters of tumor cells showing a change in morphology, usually with more eosinophilic cytoplasm, and conforming to the shape of the vascular space. In contrast, vascular pseudoinvasion (Figure 2 and Figure 3) corresponded to disaggregated tumor cells, mixed with inflammatory cells, floating in the vascular space, with no change in morphology or cytoplasmic eosinophilia. They were often large, not attached to the vessel wall, not mixed with fibrin, and there was no inflammatory reaction as that usually seen around vessels containing genuine lymphovascular emboli.

Statistical analysis was performed using Excel (Mac 16.66.1) and Biostat TGV software (https://sentiweb.fr/, accessed on 14 July 2024). A descriptive analysis of our data was performed. We also looked for the existence of statistical correlations using Fisher’s exact tests. All values of *p* < 0.05 were considered statistically significant.

## 3. Results

The clinical and histopathological characteristics of our population are summarized in Table 1.

The mean age was 58.2 years, and the median age was 59 years. The median age of patients who underwent hysterectomy for adenocarcinoma was 67 years, while patients who underwent hysterectomy for benign uterine pathology (all leiomyoma hysterectomies, with 16 of them also showing adenomyosis lesions) had a median age of 47 years. The median follow-up time for patients with adenocarcinoma was 24 months. Thirty-four (*n* = 34, 57%) patients had been operated by laparoscopy, 12 (20%) by laparotomy, and 14 (23%) by vaginal hysterectomy. An intrauterine manipulator had been used in 19 hysterectomies.

Most tumors (*n* = 26, 79%) were of low histological grade. Seven (*n* = 7, 21%) tumors were of high histological grade (grade 3). Almost half (*n* = 16, 49%) of the adenocarcinomas were MMR deficient on immunohistochemistry and showed MSI using molecular testing. Regarding the disease stage, 14 adenocarcinomas were IA, nine were IB, two were stage II, and eight were stage III. Regarding adjuvant therapy, four patients had received brachytherapy, one radiotherapy, one chemotherapy, one radiochemotherapy, three brachytherapy and chemotherapy, and seven radiotherapy and brachytherapy.

No tumor necrosis was observed in 43 (72%) tumors; nine (15%) patients had less than 10% tumor necrosis and eight (13%) tumors showed necrotic changes over more than 10% of the tumor surface. The average size of malignant tumors was 4.3 cm. The mean number of slides examined per specimen was eight slides (four slides on average for benign pathologies and 12 slides for malignant pathologies).

True vascular emboli were observed in 11 (18%) patients. Ten (91%) of these patients had been diagnosed with adenocarcinoma, while one (9%) patient presented with intravascular adenomyosis. Images of vascular pseudoinvasion were observed in 14 (23%) patients. Thirteen (93%) of these patients had adenocarcinomas and one (7%) patient was in the benign group. In the latter case, this was non-tumorous endometrium.

**Figure 4:**
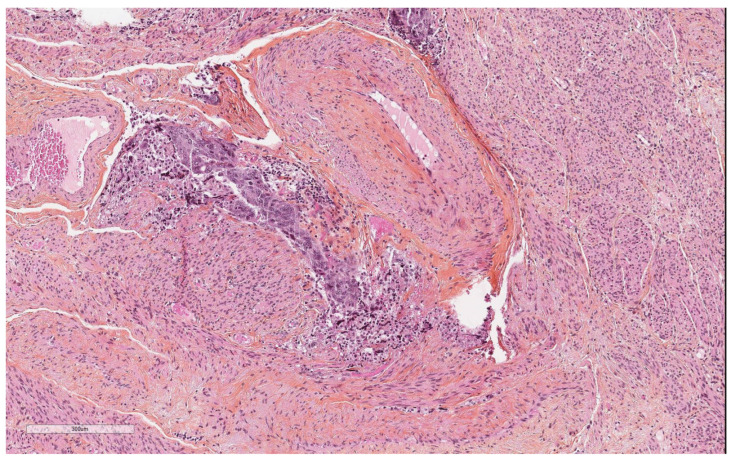
Myometrial clefts filled by tumor cells.

Regarding other artefacts, we observed tumor cells in the cervical lumen in 15 (45%) adenocarcinomas. One (3%) patient presented with detached non-tumorous endometrium in the cervix lumen. The presence of tumor cells in the tubal lumen was observed in five (15%) adenocarcinoma samples. In one (4%) benign pathology, there was non-tumorous endometrium in the tubal lumen. Clefts in the myometrium (Figure 4) were observed in 12 (36%) hysterectomies for adenocarcinoma and in four (15%) benign pathologies. In five (15%) patients with adenocarcinoma, we observed tumor cells on the uterine serosa.

Four (7%) adenocarcinoma patients relapsed during follow-up, all who presented with early-stage endometrioid adenocarcinoma; three recurred at the lymph node level and one at the cerebral level. One of these patients presented with true vascular emboli, two of these patients presented with vascular pseudoinvasion, and one patient presented with neither emboli nor vascular pseudoinvasion.

Our analysis showed that the presence of true vascular emboli in malignant cases was correlated with a higher grade (*p* = 0.041), tumor necrosis (*p* = 0.012), parametrial invasion (*p* < 0.001), and tumor cells in the lumen of the cervix (*p* = 0.008). True vascular emboli were more often present in adenocarcinoma than in benign uterine pathologies (*p* = 0.014). We did not find any statistical correlation between the presence of true vascular emboli and the FIGO stage (*p* = 0.388), the use or not of an intrauterine manipulator (*p* = 0.74), the T (*p* = 0.61) or N status (*p* = 0.64), the location of the emboli in the uterine wall (*p* = 0.46), the size of the vessels (*p* = 0.27), the venous, arterial, or lymphatic nature of the invaded vessels (*p* = 0.16), the type of surgery (*p* = 0.21), or the MSI status (*p* = 0.78). No association was observed with the artefacts studied.

The presence of vascular pseudoinvasion in malignant cases was significantly correlated with the T status (*p* = 0.004), the presence of tumor cells in the lumen of the cervix (*p* = 0.013), and the presence of myometrial clefts (*p* < 0.001). These results are summarized in Table 2.

Vascular pseudoinvasion was more often present in adenocarcinoma than in benign uterine pathologies (*p* < 0.001). We did not find any correlation between vascular pseudoinvasion and the size of the vessels concerned (*p* = 1), their location in the uterine wall (*p* = 1), the nature of the vessels (*p* = 1), the FIGO stage (*p* = 0.58), the use or not of an intrauterine manipulator (*p* = 0.19), the presence or not of necrosis (*p* = 0.19), the presence or absence of parametrial invasion (*p* = 1), the type of surgery (*p* = 0.22), the MSI status (*p* = 0.71), the maximal size of the uterus (*p* = 0.15), or the maximal size of the tumor (*p* = 0.28). A trend towards significance was observed for the histological grade (*p* = 0.06).

Regarding the other artefacts, no statistically significant correlation was seen for any of the factors studied, apart from the more frequent presence of tumor cells in the cervical lumen for malignant vs. benign pathologies (*p* < 0.001).

## 4. Discussion

With more than 8000 cases per year in France according to the 2018 National Cancer Institute (INCA) report, endometrial cancer is the fourth most frequent cancer in women, with more than 90% of these tumors corresponding to adenocarcinomas. About 80% of patients are diagnosed at an early stage with a tumor limited to the uterine corpus and a 5-year survival rate of more than 95% [18]. In 20% of cases, patients will present with lymph node metastases and/or locoregional tumor extension, and the 5-year survival rate drops to approximately 60% [19]. Surgery retains an essential place in the management of endometrial cancer, first of all to treat the disease, but also to assess the possibility of establishing adjuvant therapy. This standardized surgery includes total hysterectomy and bilateral adnexectomy, associated in some cases with lymph node dissection [20].

The presence of vascular emboli is recognized as a poor prognostic factor in endometrial adenocarcinoma, associated with a higher rate of lymph node metastases and shorter survival in these patients [21,22,23]; this criterion is included in the latest classification of the International Federation of Gynecology and Obstetrics (FIGO). In addition, in the 5th edition of the World Health Organization (WHO) report on gynecological tumors, a paragraph is dedicated to lymphovascular invasion. The authors state that vascular emboli are seen in approximately 5–15% of hysterectomies, and that artefacts due to fragmentation of poorly fixed tumors and displacement of tumor cells into vascular lumens should not be counted as genuine vascular emboli. The authors also describe a higher proportion of emboli in Mismatch Repair (MMR)-deficient cancers. In the latest recommendations for the diagnosis and treatment of endometrial cancers from the European Society for Medical Oncology (ESMO) [24], the authors differentiate between focal vascular invasion, corresponding to an invasion by tumor cells of less than four vessels, and extensive vascular invasion, when this involves more than four vessels. This is, in the latter case, a factor for poor prognosis, with more recurrences and higher mortality [9,25], while focal vascular invasion has a limited impact on prognosis [10,11,25]. The prognostic parameters in endometrial cancer can help in the decision whether or not to administer adjuvant radiotherapy [26]. In France, it is recommended for patients with stage I, grade 1 or 2 endometrial adenocarcinoma with the presence of vascular emboli, regardless of the depth of myometrial invasion. The evaluation of this parameter is therefore crucial in the therapeutic algorithm. However, like all morphological parameters in histopathology, it comes up against problems of inter-observer reproducibility [12], problems accentuated by, among other things, the frequent existence of artefacts mimicking vascular emboli or giving false images of tumor infiltration. These artefacts are represented by the retraction clefts present around the infiltrating tumor glands, by the false images of emboli corresponding to a displacement of clusters of tumor cells in the vessels, by the clefts in the myometrium, and by the presence of tumor cells on the serosa, in the lumen of the cervix, or in the tubal lumen [6,7,8,12].

Since the end of the 1980s, laparoscopic hysterectomy has become a credible alternative to hysterectomy by laparotomy. It has shown advantages such as reduced morbidity, shorter hospital stay, and better aesthetic results for the patient compared to hysterectomy by laparotomy [1,2]. About twenty years ago, Charles Ko developed this system, consisting of using an intrauterine manipulator [27] to expose the vaginal fornices and facilitate hysterectomy surgery. Nowadays, almost all laparoscopic hysterectomies are performed using an intrauterine manipulator.

Pseudoinvasion is a relatively common phenomenon in the uterus [6,7,8,12]. In some of these studies, the authors incriminated the use of an inflated intrauterine manipulator throughout the procedure. They also observed a higher rate of positive peritoneal cytology [6,7]. In response to this, many surgeons clamp the tubes and perform peritoneal cytology before inserting the intrauterine manipulator. Nevertheless, pseudoinvasion remains relatively unknown, as shown by a study carried out among Spanish gynecologists, with only 17 practitioners out of the 34 questioned being aware of it [28]. Contradictory results have been observed by some authors, showing no more vascular emboli or positive peritoneal cytology during hysterectomy by laparoscopy [9,10,11,29]. Zhang and his team [5] did not show any differences in the proportions of vascular invasion between surgeries by laparoscopy or laparotomy, but in this work the authors did not distinguish between emboli and pseudoemboli. In our series, the use of an intrauterine manipulator was not associated with emboli, pseudoemboli, or the other artefacts studied.

In our work, we found a statistical association between tumor necrosis and true vascular emboli, but no association with vascular pseudoinvasions. In some studies, the use of an intrauterine manipulator could, according to the authors, lead to a weakening of the tumor, making the tumor more likely to be fragmented and moved later in the vascular spaces by the pathologists during the step of macroscopy [8,12]. Kitahara and his team [8] observed that only polypoid tumors showed artefactual or authentic vascular invasion. We did not observe a statistical association between vascular pseudoinvasion and true vascular emboli, contrary to Folkins et al. [12]. In our work, only five patients had both emboli and pseudoemboli.

We observed more true emboli and vascular pseudoinvasions in hysterectomies for adenocarcinoma than in benign pathologies; the reasons for this difference are not clear, but one could hypothesize it is the richer cellularity/friability of the endometrial cavity in the tumoral setting. However, as shown here, hysterectomies for benign conditions have been less sampled, which could also be a bias. We also showed that the presence of true vascular emboli was correlated with grade, tumor necrosis, and parametrial invasion; thus, true emboli were well correlated with tumor aggressiveness in our study. The presence of vascular pseudoinvasion, on the other hand, was significantly correlated with other artefactual images, such as the presence of tumor cells in the cervical lumen and the presence of myometrial clefts. These findings show that the different artefacts are linked together, and could help as an additional argument in favor of pseudoinvasion.

We did not find a statistical association between the presence of true emboli or vascular pseudoinvasion and the size of the vessels involved. In the study by Kitahara et al. [8], the authors worked on a cohort of 21 low-grade, early-stage endometrial adenocarcinomas. They observed more emboli in hysterectomy by laparoscopy (33%), whereas no emboli were observed in hysterectomy by laparotomy. In addition, they observed emboli in the large vessels of the outer third of the myometrium, and none in the small vessels. It would be difficult to explain these findings solely by the use of the intrauterine manipulator; the authors rather concluded that during macroscopy, the tumor cells previously weakened by the use of the intrauterine manipulator were moved in the vessels by the blade. In our series, the eight specimens with tumor cells in the large vessels of the outer third of the myometrium also had tumor cells in the smaller vessels of the inner two-thirds of the myometrium. We did not find any statistical association between uterine/tumor size and the presence or absence of true emboli or the various artefacts studied. We did not find any statistical correlation between the presence of emboli or pseudoemboli according to the nature of the vessels invaded, contrary to what was observed in the work of Logani et al. [6], who observed a higher rate of invasion of venules and arterioles than of lymphatics. The authors suggested that the increase in intrauterine pressure could lead to a collapse of the thin walls of the lymphatics and that vascular invasion took place more easily in the venules and arterioles.

Given the small number of patients studied here, it is difficult to draw a clear conclusion for the possible prognostic role of vascular pseudoinvasion, which is a limitation of this study and of most previous studies. It would be interesting to see in the long term whether these patients present a higher risk of local or distant recurrence, a question that still remains to be answered. In cervical tumors, Ramirez and his team [30] observed reduced recurrence-free survival and overall survival in patients operated by laparoscopy compared to patients operated by laparotomy, so the authors questioned the use of the intrauterine manipulator.

## 5. Conclusions

Overall, in our series, we did not observe a statistical association between the use of an intrauterine manipulator or the type of surgery and the presence of true emboli or vascular pseudoinvasion. We nevertheless observed a statistical correlation between the images of vascular pseudoinvasions and the other artefacts analyzed, such as tumor cells in the lumen of the cervix and the myometrial clefts. In our work, we cannot clearly conclude on the responsibility of macroscopy and/or intrauterine manipulator use. Pathologists must remain vigilant and keep in mind that the phenomenon of pseudoinvasion exists in order not to misjudge vascular invasion, with the resulting prognostic and therapeutic consequences.

## Figures and Tables

**Table 1:** Clinical and histopathological data.

Variable	*n* (%)
Uterine pathology	
Endometrial adenocarcinoma	33 (55)
Benign lesion	27 (45)
MMR proteins’ status	
Deficient	16 (48.5)
Proficient	17 (51.5)
FIGO stage	
IA	14 (42.4)
IB	9 (27.3)
II	2 (6.1)
IIIA	2 (6.1)
IIIB	5 (15.2)
IIIC	1 (3.1)
pT	
pT1a	15 (45.5)
pT1b	9 (27.3)
pT2	2 (6.1)
pT3a	4 (12.1)
pT3b	3 (9.1)
pN	
pN0	31 (93.9)
pN1a	1 (3.1)
pN2	0
Not evaluable	1 (3.1)
Parametria	
Not invaded	28 (84.8)
Invaded	5 (15.2)
Type of hysterectomy	
Laparoscopy	34 (56.7)
Laparotomy	12 (20)
Vaginal	14 (23.3)
Intrauterine manipulator	
Used	19 (31.7)
Not used	41 (68.3)
Cells in the Fallopian tubes’ lumens	
Present	6 (10)
Absent	54 (90)
Cells in the lumen of the cervix	
Present	16 (26.7)
Absent	43 (71.7)
Not evaluable	1 (1.7)
Myometrial clefts	
Present	16 (26.7)
Absent	44 (73.3)
True vascular emboli	
Present	11 (18.3)
Absent	49 (81.7)
Vascular pseudoinvasion	
Present	14 (23.7)
Absent	46 (76.6)
Tumor necrosis	
Present	17 (28.3)
Absent	43 (71.7)

**Table 2:** Statistically significant correlations for vascular pseudoinvasion.

	Vascular Pseudoinvasion Present	Vascular Pseudoinvasion Absent	*p*
Cells in the cervical lumen			
Present	7	7	0.013
Absent	7	38	
Myometrial clefts			
Present	10	6	<0.001
Absent	4	40	
Uterine pathology			
Malignant	13	20	<0.001
Benign	1	26	

## Data Availability

Data available on request due to restrictions privacy.
